# Determinants and consequences of polypharmacy in patients with a depressive disorder in later life

**DOI:** 10.1111/acps.13435

**Published:** 2022-04-29

**Authors:** Carlijn Wiersema, Richard C. Oude Voshaar, Rob H. S. van den Brink, Hans Wouters, Peter Verhaak, Hannie C. Comijs, Hans W. Jeuring

**Affiliations:** ^1^ Department: University Center of Psychiatry, University of Groningen, University Medical Center Groningen Groningen The Netherlands; ^2^ Department of General Practice University of Groningen, University Medical Center Groningen Groningen The Netherlands; ^3^ Research Department, NIVEL, Netherlands Institute for Health Services Research Utrecht The Netherlands; ^4^ Department Psychiatry, Amsterdam Public Health Research Institute VU University Medical Center Amsterdam The Netherlands

**Keywords:** depression, psychopharmacology, polyphamarcy, prognosis

## Abstract

**Objectives:**

Polypharmacy and late‐life depression often congregate in the geriatric population. The primary objective is to identify determinants of polypharmacy in patients with depression, and second to examine polypharmacy in relation to various clinical phenotypes of depression and its course.

**Methods:**

A longitudinal observational study using data of the Netherlands Study of Depression in Older persons (NESDO) including 375 patients with depression ≥ 60 years and 132 non‐depressed comparisons. Linear and logistic regression were used to analyze both polypharmacy (dichotomous: ≥5 medications) and number of prescribed drugs (continuous) in relation to depression, various clinical phenotypes, and depression course.

**Results:**

Polypharmacy was more prevalent among patients with depression (46.9%) versus non‐depressed comparisons (19.7%). A lower level of education, lower cognitive functioning, and more chronic diseases were independently associated with polypharmacy. Adjusted for these determinants, polypharmacy was associated with a higher level of motivational problems, anxiety, pain, and an earlier age of onset. A higher number of drugs was associated with a worse course of late‐life depression (OR = 1.24 [95% CI: 1.03–1.49], *p* = 0.022).

**Conclusion:**

Older patients with depression have a huge risk of polypharmacy, in particular among those with an early onset depression. As an independent risk factor for chronic depression, polypharmacy needs to be identified and managed appropriately. Findings suggest that depression moderates polypharmacy through shared risk factors, including motivational problems, anxiety, and pain. The complex interaction with somatic health burden requires physicians to prescribe medications with care.

1

**Significant Outcomes**

- Polypharmacy risk is substantially increased in older patients with depression.
- Polypharmacy is an independent risk factor for chronic depression.
- Motivational problems, anxiety, and pain may increase prescribing behavior in patients with depression.

**Limitations**

- It is not known whether number of prescribed drugs is a better indicator of severity of disease than number of chronic diseases.
- The relatively small comparison group might have led to a conservative estimate of the contribution of depression to polypharmacy.

## 2 INTRODUCTION

Polypharmacy is considered a common and important problem, with a prevalence ranging from 15% up to 80% in later life.^1^ This huge variation in prevalence rates is partly because of different populations under study and different operationalization of polypharmacy.^2^ Despite the use of different definitions, polypharmacy has been consistently associated with adverse outcomes including falls, adverse drug reactions, increased length of stay in hospital, cognitive problems, and even mortality.^2^, ^3^ Although polypharmacy in older patients is thought to be especially related to an increased somatic disease burden, the presence of polypharmacy is rarely fully explained by an objective drug need.^4^ To reduce polypharmacy and its harmful effects, it is important to identify determinants that increase the risk of polypharmacy in older patients.

Several studies have identified a number of biopsychosocial determinants of polypharmacy, including age, sex, level of income, cognitive functioning, comorbidity and number of chronic diseases.^1^, ^5^, ^6^, ^7^, ^8^, ^9^ Also, a relation between polypharmacy and low social engagement,^10^ loneliness,^11^ and depressive symptoms,^7^ have been found. Mental health problems, like depression, generally go hand in hand with an objective drug need, but conversely have been associated with inappropriate drug use.^12^ Moreover, patients with depression have a more complex drug regiment when compared to non‐depressed counterparts.^13^, ^14^ Although depression is a major contributor to the global burden of diseases and, like polypharmacy, is highly prevalent in later life,^15^ the relationship between polypharmacy and late‐life depression is poorly understood.

Several course types and symptom dimensions of late‐life depression have been established.^16^, ^17^ Although some studies have found an association between polypharmacy and severity of depressive symptoms,^7^, ^11^, ^18^ other studies have found that this association disappears when properly controlled for comorbidity.^8^, ^19^ This suggests that depression may not directly affect drug consumption, but should be seen as an indicator of cumulative comorbidity which increases the risk of polypharmacy. Nonetheless, a recent study showed that primary care patients with a record of depression are more prone to polypharmacy than patients without any record of mental health problems even after controlling for use of antidepressants and somatic comorbidity.^20^

To date, polypharmacy has not been examined in a population of older patients suffering from a depression according to DSM‐ or ICD‐defined criteria. Whether the known biopsychosocial determinants of polypharmacy in older adults without depression are of similar importance in patients with a depression diagnosis is not known yet. Furthermore, knowledge is lacking whether older patients with specific subtypes of depression are more vulnerable to polypharmacy, such as an anxious, melancholic, or atypical depression. Finally, it is not known whether polypharmacy is associated with a worse course of depression. By using unique clinical data from the Netherlands Study of Depression in Older Persons (NESDO) these gaps can now be filled, which could improve the identification of polypharmacy risk among older patients with depression and ultimately may prevent further progression of negative health consequences.

### 2.1 Aims of the study

The aim of the present study is twofold. First, the association between polypharmacy and late‐life depression will be examined and explained in detail. Secondly, it is determined whether polypharmacy affects the course of late‐life depression. We first hypothesize that known biopsychosocial determinants, rather than objective drug need, explain polypharmacy in late‐life depression. Second, we hypothesize that polypharmacy in older patients with depression is associated with a poor course.

## 3 METHODS

### 3.1 Study design and sample

The study was embedded within a prospective cohort study: the Netherlands Study of Depression in Older persons (NESDO).^16^, ^21^ NESDO has included 378 depressed subjects with a major depressive disorder (95%) and/or dysthymia (26.5%) in the previous 6‐months, of which 26.5% had both disorders, and subjects with a current minor depression (5.6%). In addition, a comparison group of 132 non‐depressed comparisons were included. Depressive disorders were diagnosed at baseline and two‐year follow‐up using the Composite International Diagnostic Interview (CIDI version 2.1) according to the criteria of DSM‐IV TR. Persons with a (suspected or established) diagnosis of dementia, an organic or psychotic disorder and those with a Mini Mental State Examination‐score under 18,^22^ or insufficient mastery of the Dutch language were excluded.^21^

At baseline, data were gathered about mental health outcomes, demographic characteristics, prescribed drug use and psychosocial, biological, cognitive and genetic determinants. Interviews were performed by trained research assistants and audiotaped regularly to control for quality. Measures subject to change were evaluated again at two‐year follow‐up. At two‐year follow‐up, a total of 93/378 (24.6%) of the patients with depression and a total of 16/132 (12.1%) of the non‐depressed comparison group dropped out.^16^ In addition, each 6 months postal questionnaires were sent to the participants to study the course of depressive symptoms over time. The study protocol of NESDO was approved by the ethical review boards of the five participating mental health centres. All participants provided written informed consent.^21^

For the current study, three participants (all with a depression) had to be excluded because of missing data with respect to polypharmacy (3/510, 0.6%). Two‐year follow‐up data were available for 283/375 (75.5%) patients with depression, of which 146 (51.6%) had no past 6‐month DSM‐IV diagnosis anymore at two‐year follow‐up.

### 3.2 Measurements

#### 3.2.1 Polypharmacy

Participants were instructed to bring all prescribed medications to the interviews. Medications were registered by name, dosage and frequency of use. “Polypharmacy” was operationalized in two ways. First as the chronic simultaneous use of ≥5 medications daily, from different ATC‐codes at 3‐digit level. Dermatological preparations, medications without an ATC code, medications used less than half of the week (except drugs for which non‐daily use is common, i.e., bisphosphonates and methotrexate), and for use “if necessary” were excluded. Second, we operationalized polypharmacy as number of drugs, resulting in a continuous variable.

As the difference between the depressed and non‐depressed group might simply be because of the prescription of psychotropic drugs to treat the depressive disorder, sensitivity analyses were conducted disregarding the use of any type of psychotropic drugs, including antidepressants, benzodiazepines, antipsychotics, and mood stabilizers (including lithium).

### 3.3 Late‐life depression

#### 3.3.1 Diagnoses

At baseline and 2‐year follow‐up, the Composite International Diagnostic Interview (CIDI; WHO version 2.1) was used to assess diagnosis of the presence of an episode of major depressive disorder (MDD) and dysthymia, according to the Diagnostic and Statistical Manual of Mental Disorders‐IV‐R criteria. The CIDI is a structured clinical interview with high validity for depressive and anxiety disorders and is designed for use in research settings.^23^ Additional questions were added to diagnose a past‐month minor depression according to the research criteria of the Diagnostic and Statistical Manual of Mental Disorders‐IV.^21^

#### 3.3.2 Severity, symptom dimensions, and subtypes

The severity of depressive symptoms was assessed every 6 months by means of a self‐report questionnaire, the Inventory of Depressive Symptoms (IDS‐SR).^24^ For 28 symptoms, severity and frequency were rated on a scale from 0 to 3, adding up to total scores ranging from 0 to 84; higher scores indicating more severe depression. Factor analysis in our sample revealed three symptom dimensions, reflecting the severity of mood, motivation, and somatic symptoms of depression.^17^

With regard to the used subtypes, an *atypical symptom profile* (with mood reactivity and 2 or more of the following characteristics hyperphagia, hypersomnia, leaden paralysis, and interpersonal rejection sensitivity) was constructed by comparison of items of the IDS with DSM‐IV criteria for atypical depression following Novick's algorithm.^25^ Similarly, a *melancholic symptom profile* (with lack of mood reactivity or loss of pleasure and 3 or more of the following characteristics distinct mood quality, mood worse in morning, early morning awakening, psychomotor retardation or agitation, anorexia/weight loss, and guilt feelings) was constructed based on comparison of IDS items with DSM‐IV criteria using the algorithm proposed by Khan.^26^ Patients with depression were categorized as either having “normal” depression, depression with atypical features or depression with melancholic features. For the construction of variables regarding the severity of depressive symptoms, symptom dimensions, and subtypes, only baseline IDS‐SR scores were used.

#### 3.3.3 Course types

In accordance with Comijs et al.,^16^ five course types were distinguished on basis of the IDS‐SR scores on the five measuring points:

1. Remission, defined as at least the last two observations IDS score <14.
2. Intermittent depression, defined as at least one of the observations IDS <14 (not being the last two observations).
3. Chronic depression, defined as all IDS scores >14 and sub classified as:
  - Mild to moderate depression, defined as all IDS scores between 14 and 26.
  - Variable severity, defined as IDS scores varying between 14 and 84.
  - Moderate to severe depression, defined as all IDS scores between 26 and 84.

#### 3.3.4 Comorbid anxiety disorders and pain

In addition to assessing depressive disorders, we used the CIDI 2.1 also to assess social phobia (SP), generalized anxiety disorder (GAD), panic disorder (PD) with and without agoraphobia (AGO), and finally agoraphobia in the preceding 6 months according to DSM‐IV criteria.

Questionnaires were applied to measure the severity of anxiety and pain, as both are considered to be important dimensions in late‐life depression.^27^, ^28^, ^29^ Severity of anxiety symptoms was measured using the Beck Anxiety Inventory (BAI).^30^ The BAI is a 21 item, self‐report questionnaire primarily addressing somatic anxiety symptoms (range 0–63). A sum score of 0–9 indicates no anxiety, 10–18 mild to moderate anxiety, 19–29 moderate to severe anxiety, and 30–63 severe anxiety.^31^, ^32^ A cut‐off score of 19 is indicative of clinically relevant levels of anxiety, but other cut‐off scores have also been suggested.^33^, ^34^, ^35^ Pain intensity in the past 6 months was assessed with a visual analogue scale of 100 mm. Participants who stated to have no pain complaints received a pain intensity score of zero.

### 3.4 Determinants

Based on the literature, we examined biopsychosocial factors as potential determinants of polypharmacy in late life depression.^1^, ^5^, ^6^, ^7^, ^8^, ^9^ As social variables we included age, sex, level of education classified in elementary school, intermediate level, or higher education, as well as income. Household income was divided into three categories: ≤ 2000 euros, 2000–3800 euros, and ≥3800 per month.

Biological variables were operationalized as the number of chronic somatic diseases and global cognitive functioning. The number of chronic somatic diseases (i.e., lung disease, cardiac diseases, liver disease, atherosclerotic disease, cerebrovascular disease, diabetes mellitus, thyroid disease, malignant neoplasms, and osteoarthritis) was assessed by a self‐report questionnaire previously validated.^36^ Global cognitive functioning was assessed by the MMSE (range 0–30),^22^ which was conducted by a trained interviewer, with higher scores indicating better cognitive functioning.

With respect to psychological factors a subjective measure, loneliness, as well as an objective measure of social network size was used. Loneliness was assessed with the Loneliness Scale,^37^ a self‐report consisting of 11 dichotomized items. A score of 9 or higher is considered to indicate severe loneliness. Social network size was operationalized as the number of significant others that participants have frequent and important contact with, taking into account ‘family members, friends or close acquaintances, and only counting persons of 18 years or older who do not live in the person's household. This question had six ascending response alternatives, of which the highest four were later combined into one, resulting in the categories: “0–1” and “2–5,” and “6 or more.”

### 3.5 Statistical analyses

Differences with respect to the biopsychosocial characteristics between patients with depression and the non‐depressed comparison group at baseline were compared by Student's *t*‐tests (normally distributed continuous variables) and chi‐square tests (categorical variables). Logistic and linear regression were applied to examine differences in prevalence of polypharmacy (dependent variable in the logistic regression model) and number of prescribed drugs (dependent variable in the linear regression analyses) between the depressed and comparison group adjusted for the biopsychosocial characteristics at baseline.

Subsequently, univariate associations between demographic, biological, and social characteristics with polypharmacy were examined in the depressed subgroup only with logistic regression and with the number of prescribed drugs with linear regression. Characteristics that were significantly associated with polypharmacy and/or number of prescribed drugs were entered collectively in a multivariate model, to explore independent determinants of polypharmacy and number of prescribed drugs.

Next, we examined which characteristics of depression (specific symptom dimensions and different subtypes of depression) were associated with polypharmacy and number of prescribed drugs adjusting for the identified demographics, physical and social determinants of polypharmacy in late‐life depression. For the associative analyses on polypharmacy, sensitivity analyses were performed by excluding the use of all psychotropic drugs from the number of prescribed drugs and the polypharmacy definition.

Finally, the prognostic impact of polypharmacy and number of prescribed drugs were examined by logistic regression with the absence of any depressive disorder at two‐year follow‐up (yes/no) as the dependent variable, and adjusted for other prognostic variables, that is, age, sex, level of education, number of chronic somatic diseases, baseline depressive symptom severity, and the use of antidepressants (yes/no), benzodiazepine (yes/no), or other psychotropic drugs given for the treatment of depression. Therefore, in these models, the polypharmacy definition without the use of psychotropic drugs were considered the primary analysis. Nonetheless, a sensitivity analyses was conducted by including all psychotropic drugs into the polypharmacy definition. Similar models were build using multinomial logistic regression with the IDS‐based course type as the dependent variable (with the remitted course type as the reference).

Differences were considered statistically significant when the p‐value was less than 0.05. All analyses are conducted in SPSS version 24.

## 4 RESULTS

### 4.1 Study sample

Baseline sample characteristics of the 375 patients with depression and 132 non‐depressed comparisons are presented in Table 1. As shown, both groups did not differ with respect to age and sex, but group differences existed on all other biopsychosocial determinants. Patients with depression had a lower level of education, a lower income, more chronic somatic diseases, a lower level of cognitive functioning, higher feelings of loneliness, and a smaller network size.

**TABLE 1:** Baseline sample characteristics (*n* = 507) by depression status

Variable		Patients with depression (*n* = 375)	Non‐depressed comparisons (*n* = 132)	Statistics	*p*
*Socio‐demographics*					
Age, years	mean (SD)	70.7 (7.4)	70.1 (7.2)	*t* = −0.9, df = 505	0.380
Sex, female	*n* (%)	247 (65.9)	81 (61.4)	chi^2^ = 0.9, df = 1	0.352
Educational level				chi^2^ = 24.7, df = 2	<0.001
Low (elementary)	*n* (%)	78 (20.8)	9 (6.8)		
Intermediate	*n* (%)	219 (58.4)	71 (53.8)		
High	n (%)	78 (20.8)	52 (39.4)		
Income, euro's p/m				chi^2^ = 42.9, df = 2	<0.001
Low (<2000)	*n* (%)	205 (56.8)	40 (31.7)		
Modal (2000–3800)	*n* (%)	128 (35.5)	50 (39.7)		
High (>3800)	*n* (%)	28 (7.8)	36 (28.6)		
*Health factors*					
# chronic diseases	mean (SD)	2.1 (1.5)	1.5 (1.1)	*t* = −4.6, df = 504	<0.001
Global cognitive functioning, MMSE	mean (SD)	27.7 (2.0)	28.3 (1.6)	*T* = 3.3, df = 504	<0.001
Polypharmacy, yes	*n* (%)	176 (46.9)	26 (19.7)	chi^2^ = 30.2, df = 1	<0.001
# prescribed drugs	mean (SD)	4.7 (2.9)	2.6 (2.3)	*t* = −7.4, df = 505	<0.001
*Social factors*					
Loneliness	mean (SD)	6.1 (3.9)	1.9 (2.4)	*t* = −11.8, df = 505	<0.001
Network Size				chi^2^ = 46.1, df = 2	<0.001
0–1 person	*n* (%)	52 (14.1)	4 (3.1)		
2–5 persons	*n* (%)	172 (46.5)	30 (23.3)		
≥5 persons	*n* (%)	146 (39.5)	95 (73.6)		

*Note*: # = number of; MMSE = Mini Mental State Examination.

### 4.2 Polypharmacy

The prevalence of polypharmacy was substantially higher among patients with depression compared to non‐depressed comparisons. Moreover, the number of prescribed drugs was higher in patients with depression versus non‐depressed comparisons. A sensitivity analyses excluding the use of psychotropic drugs reduced the prevalence of polypharmacy among patients with depression to 31.5% (*n* = 118/375), which was also more than the 13.6% (*n* = 18/132) in the comparison group (chi^2^ = 15.8, df = 1, *p* < 0.001). Similarly, the mean number of drugs also remained higher among patients with depression (3.5 (SD 2.7) versus 2.4 (SD 2.1), t = −4.3, df = 505, *p* < 0.001).

When adjusted for baseline characteristics presented in Table 1, polypharmacy and the number of prescribed drugs remained associated with the presence of depression. Logistic regression revealed an odds ratio (OR) for depression of 1.88 [95% CI: 1.02–3.48] (*p* = 0.045) and linear regression showed that the presence of depression remained associated with the number of prescribed drugs (*B* (SE) = 1.46 (0.31), *β* = 0.22, *p* < 0.001). The odds‐ratio of depression on polypharmacy, however, lost significance when excluding all psychotropic drugs (OR = 1.92 [95% CI: 0.97–3.77], *p* = 0.060), whereas the association with the number of drugs remained statistically significant (*B* (SE) = 0.62 (0.28), *β* = 0.10, *p* = 0.028).

### 4.3 Determinants of polypharmacy in late‐life depression

As shown in Table 2, higher age, lower educational level, a higher number of chronic diseases, and poorer cognitive functioning were associated with polypharmacy in patients with a depression, whereas sex and social factors (loneliness and social network size) were not. The multivariate model revealed that only level of education, chronic diseases and cognitive functioning were independent determinants of polypharmacy. Results were similar with number of prescribed drugs as outcome using linear regression analyses (see Table 2).

**TABLE 2:** Determinants of polypharmacy (both as dichotomous and continuous outcome variable) in patients with depression

	Polypharmacy (yes/no)^1^				# prescribed drugs^2^			
	Univariate		Multivariate		Univariate		Multivariate	
	OR [95% CI]	p	OR [95% CI]	p	B (SE)	p	B (SE)	p
*Socio‐demographics*								
Age	1.04 [1.01–1.07]	0.006	1.02 [0.99–1.05]	0.201	0.06 (0.02)	0.006	0.02 (0.02)	0.237
Female	0.84 [0.62–1.47]	0.840			0.18 (0.32)	0.572		
Educational level								
Intermediate (vs. low)	0.68 [0.40–1.14]	0.140	1.02 [0.99–1.05]	0.600	−0.62 (0.38)	0.104	−0.25 (0.35)	0.472
High (vs. low)	0.37 [0.19–0.70]	0.003	0.43 [0.21–0.89]	0.023	−1.50 (0.46)	0.001	−1.08 (0.43)	0.012
Income								
Modal (vs. low)	0.77 [0.48–1.20]	0.249			−0.46 (0.33)	0.158		
High (vs. low)	0.86 [0.39–1.89]	0.705			−0.48 (0.58)	0.410		
*Health factors*								
# chronic diseases	1.83 [1.54–2.18]	<0.001	1.83 [1.53–2.19]	<0.001	0.77 (0.09)	<0.001	0.73 (0.09)	<0.001
Global cognitive functioning	0.81 [0.73–0.91]	<0.001	0.85 [0.75–0.96]	0.007	−0.28 (0.07)	<0.001	−0.19 (0.07)	0.008
*Social factors*								
Loneliness	1.04 [0.99–1.10]	0.112			−0.01 (0.04)	0.823		
Network size (ref = 0–1 person)								
2–5 persons	0.76 [0.41–1.41]	0.382			−0.24 (0.44)	0.582		
>5 persons	0.55 [0.29–1.05]	0.069			−0.47 (0.45)	0.302		

*Note*: # = number of; 1 = analyzed by logistic regression; 2 = analyzed by linear regression.

Sensitivity analyses, in which psychotropic drugs were excluded, revealed similar results except that low level of education was no longer significantly associated with polypharmacy in the multivariate model (*p* = 0.192). Results with respect to the number of prescribed drugs did not change meaningfully.

### 4.4 Polypharmacy and various depression phenotypes

The DSM specified subtypes of depression (atypical versus melancholic as well as major/minor depression and dysthymia) as well as DSM‐IV comorbid anxiety disorders were not consistently associated with polypharmacy or number of prescribed drugs in the fully adjusted models. Nonetheless, as shown in Table 3, we found several associations between polypharmacy and various phenotypes of depression.

**TABLE 3:** Polypharmacy in relation to various clinical phenotypes of depression

			Polypharmacy		# prescribed drugs	
Models^a^	Mean (SD)	*N* (%)	OR [95% CI]	*p*	B (SE)	*P*
*1. Severity of depressive symptoms*						
IDS sum score	30.2 (14.5)		1.02 [1.00–1.04]	0.066	0.02 (0.01)	0.110
*2. Dimensions of depressive symptoms*						
Mood subscale	8.8 (5.9)		1.00 [0.94–1.06]	0.996	−0.04 (0.04)	0.336
Motivation subscale	5.2 (3.3)		1.12 [1.01–1.23]	0.025	0.12 (0.06)	0.029
Somatic subscale	9.9 (4.4)		0.99 [0.92–1.06]	0.669	0.02 (0.04)	0.565
*3. Subtypes based on IDS*						
Atypical depression		17 (8.4)	0.68 [0.28–1.63]	0.385	−0.34 (0.49)	0.492
Melancholic depression		31 (15.3)	1.81 [0.92–3.57]	0.086	0.17 (0.40)	0.679
*4. Age of onset (MDD and dysthymia)* ^b^						
Age (continuous)	45.9 (20.9)		0.98 [0.97–0.99]	0.002	−0.02 (0.01)	0.015
Late onset (>60 years)		51 (25.2)	0.50 [0.30–0.85]	0.010	−0.63 (0.30)	0.039
*5. DSM‐IV diagnosis at baseline*						
Minor depression, past month		8 (4.0)	4.20 [0.99–17.80]	0.052	1.00 (0.85)	0.238
Major depression, past 6 months		169 (83.7)	4.36 [1.02–18.70]	0.048	1.16 (0.84)	0.168
Dysthymia, past 6 months		41 (20.3)	0.96 [1.02–18.70]	0.884	0.04 (0.32)	0.899
*6. Severity of anxiety symptoms*						
BAI sum score	18.7 (12.2)		1.03 [1.00–1.05]	0.020	0.04 (0.01)	0.006
*7. Comorbid DSM‐IV anxiety disorder*						
GAS		19 (9.4)	0.74 [0.34–1.59]	0.434	−0.69 (0.44)	0.119
Social phobia		35 (17.3)	0.91 [0.49–1.68]	0.762	−0.14 (0.36)	0.699
Panic disorder with/without agoraphobia		35 (17.3)	1.31 [0.65–2.65]	0.446	0.31 (0.41)	0.455
Agoraphobia		27 (13.4)	1.78 [0.89–3.56]	0.106	0.17 (0.42)	0.692
*8. Any DSM‐IV anxiety disorder*		*69 (34.2)*	*1.13 [0.70–1.85]*	*0.616*	*0.06 (0.29)*	*0.829*
*9. Pain*						
Severity of pain (0–100)	49.3 (25.4)		1.01 [1.00–1.02]	0.126	0.01 (0.01)	0.021

^a^ All models are adjusted for age, sex, education, MMSE, and chronic diseases.

^b^ Two separate models.

Abbreviations: BAI, Beck Anxiety Index; DSM, Diagnostic and Statistical Manual Mental Disorders; IDS, Inventory of Depressive Symptoms; MDD, major depressive disorder.

For polypharmacy (dichotomous) an association was found with the motivational subscale of the IDS (model 2), a younger age of onset (model 4), major depression at baseline (model 5), and a higher severity of anxiety symptoms (model 6). The sensitivity analyses revealed nearly similar results, except that atypical depression had significantly less often polypharmacy (OR = 0.36 [95% CI: 0.14–0.96], *p* = 0.042) and the BAI sum score was not associated with polypharmacy anymore (OR = 1.01 [95% CI: 0.99–1.03], *p* = 0.462).

The number of prescribed drugs (continuous) was associated with the motivational subscale of the IDS (model 2), a younger age of onset (model 4), a higher severity of anxiety symptoms (model 6), and a higher severity of pain (model 9). Results of the sensitivity analyses excluding psychotropic drugs revealed similar results.

### 4.5 Polypharmacy and depression course

In univariate analyses polypharmacy as well as the number of drugs prescribed was associated with non‐remission (i.e., still depressive disorder at follow‐up) (Table 4, column 1). However, after adjustment, these associations disappeared in multivariate analyses.

**TABLE 4:** Polypharmacy as determinant of chronic depression

	DSM‐defined depression		IDS‐SR based course types of depression (stable remission = reference category)							
Course type	Non‐remission		Intermittent course		Chronic course					
					Mild–moderate		Variable severity		Moderate–severe	
	OR [95% CI]	*p*	OR [95% CI]	*p*	OR [95% CI]	*p*	OR [95% CI]	*p*	OR [95% CI]	*p*
*Univariate model*										
Polypharmacy	1.99 [1.17–3.39]	0.011	1.34 [0.49–3.66]	0.562	2.48 [1.01–6.10]	0.048	3.76 [1.34–10.6]	0.012	3.95 [1.61–9.68]	0.003
# prescribed drugs	1.11 [1.01–1.22]	0.026	1.12 [0.94–1.33]	0.198	1.19 [1.01–1.39]	0.036	1.30 [1.08–1.57]	0.005	1.36 [1.16–1.60]	<0.001
Multivariate model*										
Polypharmacy	1.38 [0.74–2.55]	0.310	1.16 [0.39–3.46]	0.788	2.42 [0.90–6.52]	0.080	2.57 [0.81–8.15]	0.110	2.98 [1.08–8.24]	0.036
# prescribed drugs	1.01 [0.91–1.14]	0.788	1.07 [0.89–1.29]	0.483	1.15 [0.96–1.37]	0.119	1.17 [0.95–1.45]	0.141	1.24 [1.03–1.49]	0.022

*Note*: # = number of; * adjusted for age, sex, education, chronic diseases, baseline depression severity, and psychotropic drug use (i.e., antidepressants, benzodiazepines, antipsychotics, and mood stabilizers).

Subsequently, with regard to the different course types of depression (shown in columns 2–5 of Table 4, with “remission” course type as reference) both polypharmacy and number of prescribed drugs were associated with a more chronic course of depressive symptoms. After adjustment, both, polypharmacy and the number of prescribed drugs remained associated with severe chronic depression. Sensitivity analyses, in which the use of all types of psychotropic drugs were included in the definition of polypharmacy revealed similar results.

## 5 DISCUSSION

The most important finding of our study is that patients with depression in later life are clearly at risk of polypharmacy. Moreover, it was found that a higher number of prescribed drugs was associated with a more severe course of late‐life depression, even after adjustment for known biopsychosocial determinants of polypharmacy, including age, sex, level of education, number of chronic somatic diseases, baseline depressive symptom severity, and psychotropic drug use. This result suggest that polypharmacy is an independent risk factor for chronic depression. Among depressed older patients, a higher number of prescribed drugs was found to be associated with specific clinical phenotypes of depression, including a higher level of anxiety, pain, and motivational symptoms. Also, a younger age of onset of depression was associated with polypharmacy. To our knowledge, this is the first study that identified specific markers in older patients with depression that may help physicians to better target and manage polypharmacy.

Nearly half (47%) of depressed older patients in our study had polypharmacy, whereas in the non‐depressed comparison group only one in five older persons had polypharmacy. The higher rate of polypharmacy in late‐life depression is in accordance with previous studies.^20^ In two hospital‐based studies,^8^, ^19^ depression was positively correlated to polypharmacy, but in both studies this association was lost when corrected for the number of chronic diseases. Among a primary care population,^20^ however, polypharmacy remained significantly associated with late‐life depression when adjusted for antidepressant drug use and somatic comorbidity, which is in line with our results. These discrepant findings can be explained by the definition of polypharmacy. In our study, as in the study of Holvast and colleagues (2017), polypharmacy was defined as five or more medications, while in both hospital‐based studies polypharmacy was defined as either more than three,^19^ or four prescribed drugs.^8^ These lower thresholds for polypharmacy in the hospital‐based studies might have resulted in ceiling‐effects.

In line with previous studies, we found that polypharmacy in late‐life depression was associated with lower level of education, lower cognitive functioning, and higher number of chronic diseases.^2^, ^3^, ^7^, ^9^, ^38^, ^39^, ^40^ Our study adds to these general findings that polypharmacy was not associated with the overall severity of the depressive disorder, but with specific clinical phenotypes in patients with depression, including a higher level of motivational symptoms, anxiety, and pain. These three symptom dimensions hold a complex relationship or interaction with physical health in common.

Motivational problems (i.e., apathy) in depression are associated with functional impairment, frailty, and a lower subjective quality of well‐being.^41^, ^42^ This may lead to a greater sense of an extern locus of health control, which in turn can lead physicians to prescribing more (unnecessary) medication.^43^ A lower subjective health itself has indeed been related to polypharmacy.^4^ Secondly, motivational deficits may interfere with demanding health strategies, like changing health‐related behavior (rehabilitation, exercise, and diet). It might be that patients who lack motivation may be less interested in non‐pharmacological interventions for their psychiatric and somatic problems than those who are motivated. In these cases, clinicians may more easily chose for pharmacological strategies. A similar dynamic may apply to individuals with a low level of education but more so from the prescribers' perspective who may not have time or resources to offer non‐pharmacological interventions. These issues deserves further empirical study.

It was also found that a higher severity of anxiety symptoms, but not comorbid anxiety disorders, was associated with polypharmacy. This difference fits with the discordance between comorbid anxiety disorders and the DSM‐5 specifier for anxious distress in depression.^29^ Anxious distress in depression is associated with more functional limitations and treatment resistance,^44^, ^45^, ^46^, ^47^ and according to our results also with polypharmacy. A possible explanation is that anxious patients with depression experience more pain and somatic complaints as an expression of their depression. This may increase their medical consumption and tempt clinicians to overprescribing medication because of the somatic presentation of complaints.

Finally, a higher pain severity was associated with polypharmacy in late‐life depression, in line with previous findings in a primary care setting.^48^ The dynamic between pain and late‐life depression is complex and leads to more impaired function and more pain complaints.^49^ Pain represents a component of physical functioning and may be an independent risk factor for the onset of depression,^27^ but can also be a functional symptom of late‐life depression, often accompanied by anxiety.^50^ This faces clinicians with a problem, as pain should treated rigorously to prevent (a protracted course of) depression as well as to prevent overprescription of analgesics for functional‐depressive symptoms. A good strategy would be to attempt to taper down analgesic usage when the depressive disorder is in remission.

Finally, we demonstrated that a lower age of onset (<60 years first episode) was associated with polypharmacy among depressed older patients. At a first sight, this might be explained by a higher prevalence of somatic diseases, as depression increases the risk of somatic diseases,^51^ and an earlier onset of the depressive disorder simply increases the exposure time. Moreover, depression itself almost doubles somatic health care consumption for somatic diseases,^52^, ^53^ which may be explained by a more severe (subjective) presentation.^54^ While this explanation contrasts with the fact that the association between an earlier age of onset and polypharmacy remained significant after adjusting for the number of the somatic diseases, this might be explained by the fact that were not able to take somatic disease severity into account.

To our knowledge, the relation between polypharmacy and (the outcome of) late‐life depression has not been studied before. A higher number of prescribed drugs was associated with the most severe, chronic course of depressive symptom severity over time. Several explanations can be put forward to explain this finding. First of all, the presence of chronic diseases has been associated with a worse course of depression,^55^ also in our sample.^16^, ^56^ Nonetheless, the number of prescribed drugs remained significant when adjusted for the number of chronic diseases. A first explanation, as stated above, may point to residual confounding with the number of prescribed drugs representing a severity measure of these underlying chronic diseases. A second explanation might be adverse effects of the drugs themselves. Depression is a potential side‐effect of many drugs that are commonly prescribed to older persons, like antihypertensives, gastro‐intestinal agents and analgesics.^57^ Finally, reverse causality cannot be excluded as a protracted course of elevated depressive symptoms may be associated with an increased level of medical consumption.

## 6 LIMITATIONS

First, the relatively small comparison group and recruitment of non‐depressed participants in primary care, might have led to a conservative estimate of the contribution of depression to polypharmacy. Nonetheless, this effect is probably small as over 86% of the older population in the Netherlands regularly visits their general practitioner.^58^ Second, as a dichotomized variable for polypharmacy lowers statistical power, we also included the number of prescribed drugs as a more sensitive indicator. Third, sensitivity analyses were conducted not taking psychotropic drug use into account, to examine whether the polypharmacy was merely because of the treatment of depression. Nonetheless, when polypharmacy is studied in other diseases, for example cardiovascular disease, treatment of that disease will also be taken into account when polypharmacy is calculated (e.g., Vrettos et al., 2017).^59^ Finally, we cannot exclude the possibility that number of prescribed drugs is merely a better indicator of the severity of disease than number of somatic diseases. As pointed out above, this might indeed explain some of our findings, but is unlikely to explain all findings.

**To Conclude**: Patients with late‐life depression are more prone to polypharmacy, even when excluding psychotropic drug use and after adjusting for number of somatic diseases. The uncovered characteristics of depression associated with polypharmacy point to a complex interaction with somatic health burden. From this, it may be assumed that depression moderates polypharmacy through various risk factors of polypharmacy that coexist more in patients with depression than those without depression, such as anxiety and pain. On the one hand, these symptoms may point to a worse somatic health burden that should be treated appropriately with medications. On the other hand, increased level of medical consumption may specifically pose patients with somatic‐affective symptoms at risk for inappropriate drug use. Physicians should definitively be aware of this latter mechanism as increased drug use is associated with a poor course of depressive symptoms over time. Nonetheless, in this respect, the first mechanism is still relevant as in case of appropriate drug use, drugs with depressive side‐effects should be avoided as much as possible in somatically compromised patients with depression.

## CONFLICT OF INTEREST

All co‐authors report no conflicts of interest.

## AUTHOR CONTRIBUTIONS

**Carlijn Wiersema**, **Richard C. Oude Voshaar**, and **Hans W. Jeuring** designed the study. **Carlijn Wiersema**, **Richard C. Oude Voshaar**, and **Rob H.S. van den Brink** had full access to the data and were responsible for data integrity and statistical analyses. All authors interpreted the data. **Carlijn Wiersema**, **Richard C. Oude Voshaar**, and **Hans W. Jeuring** wrote the manuscript and integrated feedback from other authors. All authors were involved in critical revision of the study design and manuscript drafts and approved the final version.

### PEER REVIEW

The peer review history for this article is available athttps://publons.com/publon/10.1111/acps.13435.

## Data Availability

The data that support the findings of this study are available on request from the corresponding author. The data are not publicly available due to privacy or ethical restrictions.
